# Supplementary material for: Pyruvate Dehydrogenase Kinase 1 inhibition mediated oxidative phosphorylation enhancement in cartilage promotes osteoarthritis progression
Source: BMC Musculoskelet Disord. 2023 Jul 20;24:597. doi: 10.1186/s12891-023-06585-6 (PMC10357736; doi:10.1186/s12891-023-06585-6)
Supplement: Supplementary file 1 — Additional file 1. [file 12891_2023_6585_MOESM1_ESM.zip › MF.Gsea.1653627316242/GOMF_DNA_BINDING_TRANSCRIPTION_FACTOR_BINDING.html]

Details for gene set GOMF\_DNA\_BINDING\_TRANSCRIPTION\_FACTOR\_BINDING[GSEA]

|  || Dataset | 55457\_collapsed\_to\_symbols.cls.cls #OA\_versus\_normal.cls.cls #OA\_versus\_normal\_repos |
| Phenotype | cls.cls#OA\_versus\_normal\_repos |
| Upregulated in class | normal |
| GeneSet | GOMF\_DNA\_BINDING\_TRANSCRIPTION\_FACTOR\_BINDING |
| Enrichment Score (ES) | -0.3384771 |
| Normalized Enrichment Score (NES) | -1.7508966 |
| Nominal p-value | 0.0 |
| FDR q-value | 0.07450038 |
| FWER p-Value | 0.745 |
Table: GSEA Results Summary

  

Fig 1: Enrichment plot: GOMF\_DNA\_BINDING\_TRANSCRIPTION\_FACTOR\_BINDING      
 Profile of the Running ES Score & Positions of GeneSet Members on the Rank Ordered List

  

| SYMBOL | TITLE | RANK IN GENE LIST | RANK METRIC SCORE | RUNNING ES | CORE ENRICHMENT || 1 | TRIM68 | tripartite motif containing 68 [Source:HGNC Symbol;Acc:HGNC:21161] | 34 | 1.023 | 0.0046 | No |
| 2 | RNF14 | ring finger protein 14 [Source:HGNC Symbol;Acc:HGNC:10058] | 90 | 0.926 | 0.0068 | No |
| 3 | YWHAH | tyrosine 3-monooxygenase/tryptophan 5-monooxygenase activation protein eta [Source:HGNC Symbol;Acc:HGNC:12853] | 118 | 0.889 | 0.0110 | No |
| 4 | HHEX | hematopoietically expressed homeobox [Source:HGNC Symbol;Acc:HGNC:4901] | 270 | 0.774 | 0.0045 | No |
| 5 | SMAD2 | SMAD family member 2 [Source:HGNC Symbol;Acc:HGNC:6768] | 275 | 0.768 | 0.0096 | No |
| 6 | ANXA4 | annexin A4 [Source:HGNC Symbol;Acc:HGNC:542] | 300 | 0.749 | 0.0131 | No |
| 7 | RBX1 | ring-box 1 [Source:HGNC Symbol;Acc:HGNC:9928] | 348 | 0.724 | 0.0145 | No |
| 8 | SLC30A9 | solute carrier family 30 member 9 [Source:HGNC Symbol;Acc:HGNC:1329] | 390 | 0.710 | 0.0163 | No |
| 9 | DNMT3A | DNA methyltransferase 3 alpha [Source:HGNC Symbol;Acc:HGNC:2978] | 406 | 0.704 | 0.0201 | No |
| 10 | BHLHE41 | basic helix-loop-helix family member e41 [Source:HGNC Symbol;Acc:HGNC:16617] | 417 | 0.700 | 0.0243 | No |
| 11 | USF2 | "upstream transcription factor 2, c-fos interacting [Source:HGNC Symbol;Acc:HGNC:12594]" | 484 | 0.666 | 0.0238 | No |
| 12 | DHRS7B | dehydrogenase/reductase 7B [Source:HGNC Symbol;Acc:HGNC:24547] | 489 | 0.662 | 0.0282 | No |
| 13 | TRIP6 | thyroid hormone receptor interactor 6 [Source:HGNC Symbol;Acc:HGNC:12311] | 524 | 0.650 | 0.0302 | No |
| 14 | NR0B2 | nuclear receptor subfamily 0 group B member 2 [Source:HGNC Symbol;Acc:HGNC:7961] | 573 | 0.632 | 0.0308 | No |
| 15 | KAT8 | lysine acetyltransferase 8 [Source:HGNC Symbol;Acc:HGNC:17933] | 595 | 0.625 | 0.0336 | No |
| 16 | FAF1 | Fas associated factor 1 [Source:HGNC Symbol;Acc:HGNC:3578] | 686 | 0.597 | 0.0307 | No |
| 17 | FBP1 | fructose-bisphosphatase 1 [Source:HGNC Symbol;Acc:HGNC:3606] | 772 | 0.576 | 0.0280 | No |
| 18 | SRI | sorcin [Source:HGNC Symbol;Acc:HGNC:11292] | 793 | 0.570 | 0.0305 | No |
| 19 | CDKN2A | cyclin dependent kinase inhibitor 2A [Source:HGNC Symbol;Acc:HGNC:1787] | 799 | 0.570 | 0.0342 | No |
| 20 | GTF2A2 | general transcription factor IIA subunit 2 [Source:HGNC Symbol;Acc:HGNC:4647] | 899 | 0.543 | 0.0301 | No |
| 21 | ESR1 | estrogen receptor 1 [Source:HGNC Symbol;Acc:HGNC:3467] | 984 | 0.522 | 0.0271 | No |
| 22 | TRIM32 | tripartite motif containing 32 [Source:HGNC Symbol;Acc:HGNC:16380] | 993 | 0.519 | 0.0302 | No |
| 23 | NEUROD1 | neuronal differentiation 1 [Source:HGNC Symbol;Acc:HGNC:7762] | 1008 | 0.515 | 0.0328 | No |
| 24 | TFDP1 | transcription factor Dp-1 [Source:HGNC Symbol;Acc:HGNC:11749] | 1048 | 0.508 | 0.0333 | No |
| 25 | PRMT2 | protein arginine methyltransferase 2 [Source:HGNC Symbol;Acc:HGNC:5186] | 1171 | 0.482 | 0.0270 | No |
| 26 | TADA3 | transcriptional adaptor 3 [Source:HGNC Symbol;Acc:HGNC:19422] | 1295 | 0.459 | 0.0204 | No |
| 27 | GTF2B | general transcription factor IIB [Source:HGNC Symbol;Acc:HGNC:4648] | 1315 | 0.455 | 0.0221 | No |
| 28 | HDAC3 | histone deacetylase 3 [Source:HGNC Symbol;Acc:HGNC:4854] | 1325 | 0.454 | 0.0246 | No |
| 29 | WBP2 | WW domain binding protein 2 [Source:HGNC Symbol;Acc:HGNC:12738] | 1344 | 0.451 | 0.0264 | No |
| 30 | BUD31 | BUD31 homolog [Source:HGNC Symbol;Acc:HGNC:29629] | 1380 | 0.446 | 0.0268 | No |
| 31 | BBS4 | Bardet-Biedl syndrome 4 [Source:HGNC Symbol;Acc:HGNC:969] | 1404 | 0.442 | 0.0281 | No |
| 32 | PARK7 | Parkinsonism associated deglycase [Source:HGNC Symbol;Acc:HGNC:16369] | 1406 | 0.442 | 0.0312 | No |
| 33 | BCAS3 | BCAS3 microtubule associated cell migration factor [Source:HGNC Symbol;Acc:HGNC:14347] | 1471 | 0.433 | 0.0292 | No |
| 34 | BBS1 | Bardet-Biedl syndrome 1 [Source:HGNC Symbol;Acc:HGNC:966] | 1556 | 0.419 | 0.0255 | No |
| 35 | PCNA | proliferating cell nuclear antigen [Source:HGNC Symbol;Acc:HGNC:8729] | 1557 | 0.419 | 0.0285 | No |
| 36 | NAAA | N-acylethanolamine acid amidase [Source:HGNC Symbol;Acc:HGNC:736] | 1569 | 0.418 | 0.0306 | No |
| 37 | PSMD9 | "proteasome 26S subunit, non-ATPase 9 [Source:HGNC Symbol;Acc:HGNC:9567]" | 1576 | 0.417 | 0.0331 | No |
| 38 | MLX | MAX dimerization protein MLX [Source:HGNC Symbol;Acc:HGNC:11645] | 1604 | 0.413 | 0.0339 | No |
| 39 | IFI27 | interferon alpha inducible protein 27 [Source:HGNC Symbol;Acc:HGNC:5397] | 1627 | 0.409 | 0.0350 | No |
| 40 | ZNHIT3 | zinc finger HIT-type containing 3 [Source:HGNC Symbol;Acc:HGNC:12309] | 1648 | 0.407 | 0.0364 | No |
| 41 | AIP | aryl hydrocarbon receptor interacting protein [Source:HGNC Symbol;Acc:HGNC:358] | 1649 | 0.406 | 0.0393 | No |
| 42 | CNOT7 | CCR4-NOT transcription complex subunit 7 [Source:HGNC Symbol;Acc:HGNC:14101] | 1676 | 0.402 | 0.0401 | No |
| 43 | CEBPA | CCAAT enhancer binding protein alpha [Source:HGNC Symbol;Acc:HGNC:1833] | 1750 | 0.392 | 0.0370 | No |
| 44 | CSNK2B | casein kinase 2 beta [Source:HGNC Symbol;Acc:HGNC:2460] | 1788 | 0.385 | 0.0368 | No |
| 45 | C1D | C1D nuclear receptor corepressor [Source:HGNC Symbol;Acc:HGNC:29911] | 1848 | 0.379 | 0.0348 | No |
| 46 | GBX2 | gastrulation brain homeobox 2 [Source:HGNC Symbol;Acc:HGNC:4186] | 1861 | 0.378 | 0.0365 | No |
| 47 | GTF2A1 | general transcription factor IIA subunit 1 [Source:HGNC Symbol;Acc:HGNC:4646] | 1936 | 0.368 | 0.0332 | No |
| 48 | PSMC5 | "proteasome 26S subunit, ATPase 5 [Source:HGNC Symbol;Acc:HGNC:9552]" | 1987 | 0.362 | 0.0318 | No |
| 49 | NFE2L2 | "nuclear factor, erythroid 2 like 2 [Source:HGNC Symbol;Acc:HGNC:7782]" | 2034 | 0.357 | 0.0307 | No |
| 50 | SOX9 | SRY-box transcription factor 9 [Source:HGNC Symbol;Acc:HGNC:11204] | 2089 | 0.351 | 0.0289 | No |
| 51 | HSPB1 | heat shock protein family B (small) member 1 [Source:HGNC Symbol;Acc:HGNC:5246] | 2105 | 0.349 | 0.0302 | No |
| 52 | LMO2 | LIM domain only 2 [Source:HGNC Symbol;Acc:HGNC:6642] | 2122 | 0.347 | 0.0314 | No |
| 53 | SRY | sex determining region Y [Source:HGNC Symbol;Acc:HGNC:11311] | 2211 | 0.337 | 0.0268 | No |
| 54 | PITX1 | paired like homeodomain 1 [Source:HGNC Symbol;Acc:HGNC:9004] | 2263 | 0.331 | 0.0251 | No |
| 55 | CRKL | "CRK like proto-oncogene, adaptor protein [Source:HGNC Symbol;Acc:HGNC:2363]" | 2283 | 0.329 | 0.0259 | No |
| 56 | ID3 | "inhibitor of DNA binding 3, HLH protein [Source:HGNC Symbol;Acc:HGNC:5362]" | 2309 | 0.325 | 0.0262 | No |
| 57 | TRIB2 | tribbles pseudokinase 2 [Source:HGNC Symbol;Acc:HGNC:30809] | 2332 | 0.323 | 0.0268 | No |
| 58 | MAPK14 | mitogen-activated protein kinase 14 [Source:HGNC Symbol;Acc:HGNC:6876] | 2352 | 0.320 | 0.0275 | No |
| 59 | UBXN7 | UBX domain protein 7 [Source:HGNC Symbol;Acc:HGNC:29119] | 2354 | 0.320 | 0.0297 | No |
| 60 | ISL1 | ISL LIM homeobox 1 [Source:HGNC Symbol;Acc:HGNC:6132] | 2373 | 0.318 | 0.0306 | No |
| 61 | CHCHD2 | coiled-coil-helix-coiled-coil-helix domain containing 2 [Source:HGNC Symbol;Acc:HGNC:21645] | 2431 | 0.310 | 0.0282 | No |
| 62 | DGKQ | diacylglycerol kinase theta [Source:HGNC Symbol;Acc:HGNC:2856] | 2516 | 0.302 | 0.0237 | No |
| 63 | PKN1 | protein kinase N1 [Source:HGNC Symbol;Acc:HGNC:9405] | 2540 | 0.298 | 0.0239 | No |
| 64 | NCOR1 | nuclear receptor corepressor 1 [Source:HGNC Symbol;Acc:HGNC:7672] | 2549 | 0.297 | 0.0254 | No |
| 65 | MED12 | mediator complex subunit 12 [Source:HGNC Symbol;Acc:HGNC:11957] | 2588 | 0.292 | 0.0245 | No |
| 66 | BBS10 | Bardet-Biedl syndrome 10 [Source:HGNC Symbol;Acc:HGNC:26291] | 2688 | 0.280 | 0.0186 | No |
| 67 | NIF3L1 | NGG1 interacting factor 3 like 1 [Source:HGNC Symbol;Acc:HGNC:13390] | 2807 | 0.269 | 0.0110 | No |
| 68 | PADI2 | peptidyl arginine deiminase 2 [Source:HGNC Symbol;Acc:HGNC:18341] | 2810 | 0.269 | 0.0128 | No |
| 69 | KAT6B | lysine acetyltransferase 6B [Source:HGNC Symbol;Acc:HGNC:17582] | 2827 | 0.267 | 0.0134 | No |
| 70 | BCOR | BCL6 corepressor [Source:HGNC Symbol;Acc:HGNC:20893] | 2890 | 0.262 | 0.0103 | No |
| 71 | MAGEA2 | MAGE family member A2 [Source:HGNC Symbol;Acc:HGNC:6800] | 2952 | 0.255 | 0.0073 | No |
| 72 | GATA4 | GATA binding protein 4 [Source:HGNC Symbol;Acc:HGNC:4173] | 3009 | 0.249 | 0.0046 | No |
| 73 | NFATC1 | nuclear factor of activated T cells 1 [Source:HGNC Symbol;Acc:HGNC:7775] | 3027 | 0.247 | 0.0050 | No |
| 74 | TAF7 | TATA-box binding protein associated factor 7 [Source:HGNC Symbol;Acc:HGNC:11541] | 3035 | 0.246 | 0.0062 | No |
| 75 | GAS2L1 | growth arrest specific 2 like 1 [Source:HGNC Symbol;Acc:HGNC:16955] | 3136 | 0.237 | -0.0001 | No |
| 76 | PTPRT | protein tyrosine phosphatase receptor type T [Source:HGNC Symbol;Acc:HGNC:9682] | 3157 | 0.234 | -0.0001 | No |
| 77 | PRAME | PRAME nuclear receptor transcriptional regulator [Source:HGNC Symbol;Acc:HGNC:9336] | 3235 | 0.227 | -0.0046 | No |
| 78 | MED24 | mediator complex subunit 24 [Source:HGNC Symbol;Acc:HGNC:22963] | 3246 | 0.226 | -0.0038 | No |
| 79 | DACT1 | dishevelled binding antagonist of beta catenin 1 [Source:HGNC Symbol;Acc:HGNC:17748] | 3275 | 0.223 | -0.0044 | No |
| 80 | LRIF1 | ligand dependent nuclear receptor interacting factor 1 [Source:HGNC Symbol;Acc:HGNC:30299] | 3284 | 0.222 | -0.0035 | No |
| 81 | ELK1 | ETS transcription factor ELK1 [Source:HGNC Symbol;Acc:HGNC:3321] | 3357 | 0.216 | -0.0077 | No |
| 82 | PARP1 | poly(ADP-ribose) polymerase 1 [Source:HGNC Symbol;Acc:HGNC:270] | 3410 | 0.210 | -0.0104 | No |
| 83 | MDFIC | MyoD family inhibitor domain containing [Source:HGNC Symbol;Acc:HGNC:28870] | 3419 | 0.209 | -0.0095 | No |
| 84 | LMO4 | LIM domain only 4 [Source:HGNC Symbol;Acc:HGNC:6644] | 3508 | 0.202 | -0.0151 | No |
| 85 | ARNT2 | aryl hydrocarbon receptor nuclear translocator 2 [Source:HGNC Symbol;Acc:HGNC:16876] | 3512 | 0.201 | -0.0139 | No |
| 86 | RBFOX2 | RNA binding fox-1 homolog 2 [Source:HGNC Symbol;Acc:HGNC:9906] | 3523 | 0.200 | -0.0133 | No |
| 87 | VDR | vitamin D receptor [Source:HGNC Symbol;Acc:HGNC:12679] | 3571 | 0.197 | -0.0156 | No |
| 88 | TGFB1I1 | transforming growth factor beta 1 induced transcript 1 [Source:HGNC Symbol;Acc:HGNC:11767] | 3628 | 0.191 | -0.0187 | No |
| 89 | PRRX1 | paired related homeobox 1 [Source:HGNC Symbol;Acc:HGNC:9142] | 3657 | 0.189 | -0.0196 | No |
| 90 | RBBP8 | "RB binding protein 8, endonuclease [Source:HGNC Symbol;Acc:HGNC:9891]" | 3739 | 0.182 | -0.0248 | No |
| 91 | MAX | MYC associated factor X [Source:HGNC Symbol;Acc:HGNC:6913] | 3804 | 0.176 | -0.0287 | No |
| 92 | HMGN3 | high mobility group nucleosomal binding domain 3 [Source:HGNC Symbol;Acc:HGNC:12312] | 3818 | 0.174 | -0.0285 | No |
| 93 | CNOT1 | CCR4-NOT transcription complex subunit 1 [Source:HGNC Symbol;Acc:HGNC:7877] | 3842 | 0.172 | -0.0291 | No |
| 94 | TAF12 | TATA-box binding protein associated factor 12 [Source:HGNC Symbol;Acc:HGNC:11545] | 3843 | 0.172 | -0.0279 | No |
| 95 | ADD1 | adducin 1 [Source:HGNC Symbol;Acc:HGNC:243] | 3872 | 0.167 | -0.0289 | No |
| 96 | PBX1 | PBX homeobox 1 [Source:HGNC Symbol;Acc:HGNC:8632] | 3887 | 0.166 | -0.0288 | No |
| 97 | RELA | "RELA proto-oncogene, NF-kB subunit [Source:HGNC Symbol;Acc:HGNC:9955]" | 3942 | 0.161 | -0.0320 | No |
| 98 | NR1I2 | nuclear receptor subfamily 1 group I member 2 [Source:HGNC Symbol;Acc:HGNC:7968] | 3956 | 0.160 | -0.0319 | No |
| 99 | ZFPM2 | "zinc finger protein, FOG family member 2 [Source:HGNC Symbol;Acc:HGNC:16700]" | 3958 | 0.160 | -0.0308 | No |
| 100 | RUNX2 | RUNX family transcription factor 2 [Source:HGNC Symbol;Acc:HGNC:10472] | 3993 | 0.157 | -0.0324 | No |
| 101 | HEY2 | hes related family bHLH transcription factor with YRPW motif 2 [Source:HGNC Symbol;Acc:HGNC:4881] | 4058 | 0.150 | -0.0365 | No |
| 102 | SMAD3 | SMAD family member 3 [Source:HGNC Symbol;Acc:HGNC:6769] | 4061 | 0.150 | -0.0356 | No |
| 103 | KEAP1 | kelch like ECH associated protein 1 [Source:HGNC Symbol;Acc:HGNC:23177] | 4095 | 0.146 | -0.0372 | No |
| 104 | TRIP4 | thyroid hormone receptor interactor 4 [Source:HGNC Symbol;Acc:HGNC:12310] | 4105 | 0.146 | -0.0368 | No |
| 105 | FHL2 | four and a half LIM domains 2 [Source:HGNC Symbol;Acc:HGNC:3703] | 4127 | 0.143 | -0.0375 | No |
| 106 | NBN | nibrin [Source:HGNC Symbol;Acc:HGNC:7652] | 4136 | 0.143 | -0.0371 | No |
| 107 | VHL | von Hippel-Lindau tumor suppressor [Source:HGNC Symbol;Acc:HGNC:12687] | 4142 | 0.143 | -0.0365 | No |
| 108 | MKKS | MKKS centrosomal shuttling protein [Source:HGNC Symbol;Acc:HGNC:7108] | 4182 | 0.140 | -0.0386 | No |
| 109 | WIPI1 | "WD repeat domain, phosphoinositide interacting 1 [Source:HGNC Symbol;Acc:HGNC:25471]" | 4188 | 0.139 | -0.0380 | No |
| 110 | MEF2C | myocyte enhancer factor 2C [Source:HGNC Symbol;Acc:HGNC:6996] | 4218 | 0.135 | -0.0394 | No |
| 111 | SMARCE1 | "SWI/SNF related, matrix associated, actin dependent regulator of chromatin, subfamily e, member 1 [Source:HGNC Symbol;Acc:HGNC:11109]" | 4255 | 0.132 | -0.0413 | No |
| 112 | TAF6 | TATA-box binding protein associated factor 6 [Source:HGNC Symbol;Acc:HGNC:11540] | 4260 | 0.132 | -0.0407 | No |
| 113 | NCOA2 | nuclear receptor coactivator 2 [Source:HGNC Symbol;Acc:HGNC:7669] | 4284 | 0.129 | -0.0416 | No |
| 114 | PROX1 | prospero homeobox 1 [Source:HGNC Symbol;Acc:HGNC:9459] | 4299 | 0.128 | -0.0418 | No |
| 115 | TCF21 | transcription factor 21 [Source:HGNC Symbol;Acc:HGNC:11632] | 4321 | 0.127 | -0.0426 | No |
| 116 | HDAC5 | histone deacetylase 5 [Source:HGNC Symbol;Acc:HGNC:14068] | 4336 | 0.125 | -0.0428 | No |
| 117 | CD34 | CD34 molecule [Source:HGNC Symbol;Acc:HGNC:1662] | 4341 | 0.125 | -0.0422 | No |
| 118 | PHB2 | prohibitin 2 [Source:HGNC Symbol;Acc:HGNC:30306] | 4354 | 0.124 | -0.0423 | No |
| 119 | NFYB | nuclear transcription factor Y subunit beta [Source:HGNC Symbol;Acc:HGNC:7805] | 4389 | 0.122 | -0.0442 | No |
| 120 | CENPF | centromere protein F [Source:HGNC Symbol;Acc:HGNC:1857] | 4437 | 0.118 | -0.0471 | No |
| 121 | STAT1 | signal transducer and activator of transcription 1 [Source:HGNC Symbol;Acc:HGNC:11362] | 4495 | 0.113 | -0.0508 | No |
| 122 | SETD3 | "SET domain containing 3, actin histidine methyltransferase [Source:HGNC Symbol;Acc:HGNC:20493]" | 4506 | 0.113 | -0.0508 | No |
| 123 | PURA | purine rich element binding protein A [Source:HGNC Symbol;Acc:HGNC:9701] | 4514 | 0.112 | -0.0506 | No |
| 124 | PRPF6 | pre-mRNA processing factor 6 [Source:HGNC Symbol;Acc:HGNC:15860] | 4567 | 0.108 | -0.0540 | No |
| 125 | FLT3 | fms related receptor tyrosine kinase 3 [Source:HGNC Symbol;Acc:HGNC:3765] | 4657 | 0.099 | -0.0604 | No |
| 126 | TBX5 | T-box transcription factor 5 [Source:HGNC Symbol;Acc:HGNC:11604] | 4748 | 0.091 | -0.0669 | No |
| 127 | TBX3 | T-box transcription factor 3 [Source:HGNC Symbol;Acc:HGNC:11602] | 4790 | 0.088 | -0.0696 | No |
| 128 | OASL | 2'-5'-oligoadenylate synthetase like [Source:HGNC Symbol;Acc:HGNC:8090] | 4812 | 0.086 | -0.0707 | No |
| 129 | WWP2 | WW domain containing E3 ubiquitin protein ligase 2 [Source:HGNC Symbol;Acc:HGNC:16804] | 4818 | 0.086 | -0.0704 | No |
| 130 | H2BC9 | H2B clustered histone 9 [Source:HGNC Symbol;Acc:HGNC:4755] | 4859 | 0.083 | -0.0731 | No |
| 131 | KAT2B | lysine acetyltransferase 2B [Source:HGNC Symbol;Acc:HGNC:8638] | 4867 | 0.082 | -0.0730 | No |
| 132 | HCFC1 | host cell factor C1 [Source:HGNC Symbol;Acc:HGNC:4839] | 4937 | 0.077 | -0.0780 | No |
| 133 | TP53BP1 | tumor protein p53 binding protein 1 [Source:HGNC Symbol;Acc:HGNC:11999] | 4946 | 0.076 | -0.0781 | No |
| 134 | CBX5 | chromobox 5 [Source:HGNC Symbol;Acc:HGNC:1555] | 4963 | 0.075 | -0.0788 | No |
| 135 | PPID | peptidylprolyl isomerase D [Source:HGNC Symbol;Acc:HGNC:9257] | 5029 | 0.071 | -0.0835 | No |
| 136 | TCF7L2 | transcription factor 7 like 2 [Source:HGNC Symbol;Acc:HGNC:11641] | 5034 | 0.070 | -0.0834 | No |
| 137 | SMARCD3 | "SWI/SNF related, matrix associated, actin dependent regulator of chromatin, subfamily d, member 3 [Source:HGNC Symbol;Acc:HGNC:11108]" | 5036 | 0.070 | -0.0829 | No |
| 138 | YAP1 | Yes1 associated transcriptional regulator [Source:HGNC Symbol;Acc:HGNC:16262] | 5041 | 0.070 | -0.0828 | No |
| 139 | CTBP2 | C-terminal binding protein 2 [Source:HGNC Symbol;Acc:HGNC:2495] | 5066 | 0.067 | -0.0842 | No |
| 140 | E2F1 | E2F transcription factor 1 [Source:HGNC Symbol;Acc:HGNC:3113] | 5137 | 0.061 | -0.0894 | No |
| 141 | NSD1 | nuclear receptor binding SET domain protein 1 [Source:HGNC Symbol;Acc:HGNC:14234] | 5178 | 0.058 | -0.0921 | No |
| 142 | HDAC9 | histone deacetylase 9 [Source:HGNC Symbol;Acc:HGNC:14065] | 5183 | 0.058 | -0.0921 | No |
| 143 | TP73 | tumor protein p73 [Source:HGNC Symbol;Acc:HGNC:12003] | 5222 | 0.055 | -0.0947 | No |
| 144 | FOXC1 | forkhead box C1 [Source:HGNC Symbol;Acc:HGNC:3800] | 5227 | 0.054 | -0.0946 | No |
| 145 | MED17 | mediator complex subunit 17 [Source:HGNC Symbol;Acc:HGNC:2375] | 5257 | 0.052 | -0.0966 | No |
| 146 | HMGB2 | high mobility group box 2 [Source:HGNC Symbol;Acc:HGNC:5000] | 5272 | 0.050 | -0.0974 | No |
| 147 | TAL1 | "TAL bHLH transcription factor 1, erythroid differentiation factor [Source:HGNC Symbol;Acc:HGNC:11556]" | 5406 | 0.040 | -0.1077 | No |
| 148 | NR1H2 | nuclear receptor subfamily 1 group H member 2 [Source:HGNC Symbol;Acc:HGNC:7965] | 5413 | 0.039 | -0.1079 | No |
| 149 | SNW1 | SNW domain containing 1 [Source:HGNC Symbol;Acc:HGNC:16696] | 5472 | 0.035 | -0.1123 | No |
| 150 | RUNX1T1 | RUNX1 partner transcriptional co-repressor 1 [Source:HGNC Symbol;Acc:HGNC:1535] | 5503 | 0.032 | -0.1145 | No |
| 151 | TP53 | tumor protein p53 [Source:HGNC Symbol;Acc:HGNC:11998] | 5511 | 0.032 | -0.1148 | No |
| 152 | GATA6 | GATA binding protein 6 [Source:HGNC Symbol;Acc:HGNC:4174] | 5537 | 0.030 | -0.1166 | No |
| 153 | STAT5B | signal transducer and activator of transcription 5B [Source:HGNC Symbol;Acc:HGNC:11367] | 5554 | 0.028 | -0.1177 | No |
| 154 | GFI1B | growth factor independent 1B transcriptional repressor [Source:HGNC Symbol;Acc:HGNC:4238] | 5591 | 0.025 | -0.1204 | No |
| 155 | BCL2 | BCL2 apoptosis regulator [Source:HGNC Symbol;Acc:HGNC:990] | 5609 | 0.023 | -0.1216 | No |
| 156 | MEF2A | myocyte enhancer factor 2A [Source:HGNC Symbol;Acc:HGNC:6993] | 5628 | 0.022 | -0.1229 | No |
| 157 | HOXA7 | homeobox A7 [Source:HGNC Symbol;Acc:HGNC:5108] | 5634 | 0.021 | -0.1231 | No |
| 158 | NCAPG2 | non-SMC condensin II complex subunit G2 [Source:HGNC Symbol;Acc:HGNC:21904] | 5700 | 0.016 | -0.1282 | No |
| 159 | SMARCA1 | "SWI/SNF related, matrix associated, actin dependent regulator of chromatin, subfamily a, member 1 [Source:HGNC Symbol;Acc:HGNC:11097]" | 5723 | 0.014 | -0.1299 | No |
| 160 | CEBPG | CCAAT enhancer binding protein gamma [Source:HGNC Symbol;Acc:HGNC:1837] | 5734 | 0.013 | -0.1306 | No |
| 161 | ARID1A | AT-rich interaction domain 1A [Source:HGNC Symbol;Acc:HGNC:11110] | 5735 | 0.013 | -0.1305 | No |
| 162 | YWHAZ | tyrosine 3-monooxygenase/tryptophan 5-monooxygenase activation protein zeta [Source:HGNC Symbol;Acc:HGNC:12855] | 5770 | 0.010 | -0.1331 | No |
| 163 | KAT6A | lysine acetyltransferase 6A [Source:HGNC Symbol;Acc:HGNC:13013] | 5778 | 0.010 | -0.1336 | No |
| 164 | MED14 | mediator complex subunit 14 [Source:HGNC Symbol;Acc:HGNC:2370] | 5867 | 0.004 | -0.1406 | No |
| 165 | PPARG | peroxisome proliferator activated receptor gamma [Source:HGNC Symbol;Acc:HGNC:9236] | 6006 | -0.005 | -0.1516 | No |
| 166 | RB1 | RB transcriptional corepressor 1 [Source:HGNC Symbol;Acc:HGNC:9884] | 6013 | -0.006 | -0.1521 | No |
| 167 | FOXL2 | forkhead box L2 [Source:HGNC Symbol;Acc:HGNC:1092] | 6068 | -0.009 | -0.1563 | No |
| 168 | PPARGC1B | PPARG coactivator 1 beta [Source:HGNC Symbol;Acc:HGNC:30022] | 6184 | -0.019 | -0.1654 | No |
| 169 | ANKRD2 | ankyrin repeat domain 2 [Source:HGNC Symbol;Acc:HGNC:495] | 6186 | -0.019 | -0.1653 | No |
| 170 | MED16 | mediator complex subunit 16 [Source:HGNC Symbol;Acc:HGNC:17556] | 6240 | -0.023 | -0.1694 | No |
| 171 | SIRT2 | sirtuin 2 [Source:HGNC Symbol;Acc:HGNC:10886] | 6279 | -0.026 | -0.1723 | No |
| 172 | TAF9 | TATA-box binding protein associated factor 9 [Source:HGNC Symbol;Acc:HGNC:11542] | 6297 | -0.027 | -0.1734 | No |
| 173 | KAT5 | lysine acetyltransferase 5 [Source:HGNC Symbol;Acc:HGNC:5275] | 6379 | -0.034 | -0.1797 | No |
| 174 | TCF12 | transcription factor 12 [Source:HGNC Symbol;Acc:HGNC:11623] | 6398 | -0.035 | -0.1809 | No |
| 175 | ANKRD1 | ankyrin repeat domain 1 [Source:HGNC Symbol;Acc:HGNC:15819] | 6416 | -0.037 | -0.1820 | No |
| 176 | PBXIP1 | PBX homeobox interacting protein 1 [Source:HGNC Symbol;Acc:HGNC:21199] | 6434 | -0.038 | -0.1831 | No |
| 177 | PRKCB | protein kinase C beta [Source:HGNC Symbol;Acc:HGNC:9395] | 6453 | -0.039 | -0.1842 | No |
| 178 | BBS7 | Bardet-Biedl syndrome 7 [Source:HGNC Symbol;Acc:HGNC:18758] | 6460 | -0.039 | -0.1844 | No |
| 179 | LHX3 | LIM homeobox 3 [Source:HGNC Symbol;Acc:HGNC:6595] | 6495 | -0.042 | -0.1869 | No |
| 180 | NCOR2 | nuclear receptor corepressor 2 [Source:HGNC Symbol;Acc:HGNC:7673] | 6497 | -0.042 | -0.1866 | No |
| 181 | CDK5RAP3 | CDK5 regulatory subunit associated protein 3 [Source:HGNC Symbol;Acc:HGNC:18673] | 6583 | -0.048 | -0.1931 | No |
| 182 | GTF2I | general transcription factor IIi [Source:HGNC Symbol;Acc:HGNC:4659] | 6588 | -0.049 | -0.1931 | No |
| 183 | BRMS1 | BRMS1 transcriptional repressor and anoikis regulator [Source:HGNC Symbol;Acc:HGNC:17262] | 6602 | -0.050 | -0.1937 | No |
| 184 | PSMA6 | proteasome 20S subunit alpha 6 [Source:HGNC Symbol;Acc:HGNC:9535] | 6721 | -0.057 | -0.2028 | No |
| 185 | MMS19 | "MMS19 homolog, cytosolic iron-sulfur assembly component [Source:HGNC Symbol;Acc:HGNC:13824]" | 6733 | -0.058 | -0.2032 | No |
| 186 | HMGA2 | high mobility group AT-hook 2 [Source:HGNC Symbol;Acc:HGNC:5009] | 6850 | -0.067 | -0.2121 | No |
| 187 | ZNF516 | zinc finger protein 516 [Source:HGNC Symbol;Acc:HGNC:28990] | 6879 | -0.070 | -0.2138 | No |
| 188 | NR0B1 | nuclear receptor subfamily 0 group B member 1 [Source:HGNC Symbol;Acc:HGNC:7960] | 6885 | -0.070 | -0.2137 | No |
| 189 | SRC | "SRC proto-oncogene, non-receptor tyrosine kinase [Source:HGNC Symbol;Acc:HGNC:11283]" | 6940 | -0.075 | -0.2175 | No |
| 190 | JMJD1C | jumonji domain containing 1C [Source:HGNC Symbol;Acc:HGNC:12313] | 6989 | -0.079 | -0.2208 | No |
| 191 | SP3 | Sp3 transcription factor [Source:HGNC Symbol;Acc:HGNC:11208] | 7033 | -0.082 | -0.2236 | No |
| 192 | FOXP3 | forkhead box P3 [Source:HGNC Symbol;Acc:HGNC:6106] | 7035 | -0.082 | -0.2231 | No |
| 193 | CITED2 | Cbp/p300 interacting transactivator with Glu/Asp rich carboxy-terminal domain 2 [Source:HGNC Symbol;Acc:HGNC:1987] | 7100 | -0.087 | -0.2276 | No |
| 194 | HNF4A | hepatocyte nuclear factor 4 alpha [Source:HGNC Symbol;Acc:HGNC:5024] | 7160 | -0.093 | -0.2316 | No |
| 195 | NCOA6 | nuclear receptor coactivator 6 [Source:HGNC Symbol;Acc:HGNC:15936] | 7171 | -0.093 | -0.2318 | No |
| 196 | MED4 | mediator complex subunit 4 [Source:HGNC Symbol;Acc:HGNC:17903] | 7236 | -0.098 | -0.2362 | No |
| 197 | CALR | calreticulin [Source:HGNC Symbol;Acc:HGNC:1455] | 7291 | -0.102 | -0.2398 | No |
| 198 | NLK | nemo like kinase [Source:HGNC Symbol;Acc:HGNC:29858] | 7336 | -0.105 | -0.2426 | No |
| 199 | SKI | SKI proto-oncogene [Source:HGNC Symbol;Acc:HGNC:10896] | 7349 | -0.106 | -0.2428 | No |
| 200 | ASCL1 | achaete-scute family bHLH transcription factor 1 [Source:HGNC Symbol;Acc:HGNC:738] | 7375 | -0.108 | -0.2440 | No |
| 201 | RXRA | retinoid X receptor alpha [Source:HGNC Symbol;Acc:HGNC:10477] | 7381 | -0.109 | -0.2436 | No |
| 202 | DCAF13 | DDB1 and CUL4 associated factor 13 [Source:HGNC Symbol;Acc:HGNC:24535] | 7459 | -0.115 | -0.2490 | No |
| 203 | LEF1 | lymphoid enhancer binding factor 1 [Source:HGNC Symbol;Acc:HGNC:6551] | 7467 | -0.116 | -0.2487 | No |
| 204 | CREB1 | cAMP responsive element binding protein 1 [Source:HGNC Symbol;Acc:HGNC:2345] | 7470 | -0.116 | -0.2480 | No |
| 205 | TCF3 | transcription factor 3 [Source:HGNC Symbol;Acc:HGNC:11633] | 7521 | -0.120 | -0.2512 | No |
| 206 | TAF4B | TATA-box binding protein associated factor 4b [Source:HGNC Symbol;Acc:HGNC:11538] | 7596 | -0.127 | -0.2562 | No |
| 207 | NHLH2 | nescient helix-loop-helix 2 [Source:HGNC Symbol;Acc:HGNC:7818] | 7626 | -0.129 | -0.2576 | No |
| 208 | HCLS1 | hematopoietic cell-specific Lyn substrate 1 [Source:HGNC Symbol;Acc:HGNC:4844] | 7653 | -0.131 | -0.2587 | No |
| 209 | POU1F1 | POU class 1 homeobox 1 [Source:HGNC Symbol;Acc:HGNC:9210] | 7681 | -0.133 | -0.2599 | No |
| 210 | HOXC13 | homeobox C13 [Source:HGNC Symbol;Acc:HGNC:5125] | 7738 | -0.137 | -0.2634 | No |
| 211 | SOX10 | SRY-box transcription factor 10 [Source:HGNC Symbol;Acc:HGNC:11190] | 7788 | -0.141 | -0.2664 | No |
| 212 | HIF1AN | hypoxia inducible factor 1 subunit alpha inhibitor [Source:HGNC Symbol;Acc:HGNC:17113] | 7815 | -0.142 | -0.2674 | No |
| 213 | CRTC3 | CREB regulated transcription coactivator 3 [Source:HGNC Symbol;Acc:HGNC:26148] | 7838 | -0.143 | -0.2682 | No |
| 214 | MYOD1 | myogenic differentiation 1 [Source:HGNC Symbol;Acc:HGNC:7611] | 7839 | -0.143 | -0.2671 | No |
| 215 | GMNN | geminin DNA replication inhibitor [Source:HGNC Symbol;Acc:HGNC:17493] | 7866 | -0.146 | -0.2682 | No |
| 216 | HDAC1 | histone deacetylase 1 [Source:HGNC Symbol;Acc:HGNC:4852] | 7911 | -0.148 | -0.2706 | No |
| 217 | TLE4 | "TLE family member 4, transcriptional corepressor [Source:HGNC Symbol;Acc:HGNC:11840]" | 7915 | -0.149 | -0.2698 | No |
| 218 | TFDP3 | transcription factor Dp family member 3 [Source:HGNC Symbol;Acc:HGNC:24603] | 7920 | -0.149 | -0.2691 | No |
| 219 | TAF4 | TATA-box binding protein associated factor 4 [Source:HGNC Symbol;Acc:HGNC:11537] | 7966 | -0.153 | -0.2716 | No |
| 220 | NLRP3 | NLR family pyrin domain containing 3 [Source:HGNC Symbol;Acc:HGNC:16400] | 8014 | -0.156 | -0.2742 | No |
| 221 | CREM | cAMP responsive element modulator [Source:HGNC Symbol;Acc:HGNC:2352] | 8026 | -0.157 | -0.2740 | No |
| 222 | PITX2 | paired like homeodomain 2 [Source:HGNC Symbol;Acc:HGNC:9005] | 8137 | -0.166 | -0.2816 | No |
| 223 | ID4 | "inhibitor of DNA binding 4, HLH protein [Source:HGNC Symbol;Acc:HGNC:5363]" | 8174 | -0.169 | -0.2833 | No |
| 224 | HAND2 | heart and neural crest derivatives expressed 2 [Source:HGNC Symbol;Acc:HGNC:4808] | 8211 | -0.172 | -0.2849 | No |
| 225 | HEY1 | hes related family bHLH transcription factor with YRPW motif 1 [Source:HGNC Symbol;Acc:HGNC:4880] | 8213 | -0.172 | -0.2838 | No |
| 226 | AR | androgen receptor [Source:HGNC Symbol;Acc:HGNC:644] | 8217 | -0.173 | -0.2828 | No |
| 227 | KDM3A | lysine demethylase 3A [Source:HGNC Symbol;Acc:HGNC:20815] | 8264 | -0.176 | -0.2852 | No |
| 228 | TRIP12 | thyroid hormone receptor interactor 12 [Source:HGNC Symbol;Acc:HGNC:12306] | 8290 | -0.178 | -0.2859 | No |
| 229 | WFS1 | wolframin ER transmembrane glycoprotein [Source:HGNC Symbol;Acc:HGNC:12762] | 8299 | -0.178 | -0.2853 | No |
| 230 | TP53BP2 | tumor protein p53 binding protein 2 [Source:HGNC Symbol;Acc:HGNC:12000] | 8336 | -0.181 | -0.2869 | No |
| 231 | HDAC4 | histone deacetylase 4 [Source:HGNC Symbol;Acc:HGNC:14063] | 8416 | -0.187 | -0.2919 | No |
| 232 | DUSP26 | dual specificity phosphatase 26 [Source:HGNC Symbol;Acc:HGNC:28161] | 8424 | -0.187 | -0.2911 | No |
| 233 | STAT3 | signal transducer and activator of transcription 3 [Source:HGNC Symbol;Acc:HGNC:11364] | 8453 | -0.190 | -0.2920 | No |
| 234 | NR1H4 | nuclear receptor subfamily 1 group H member 4 [Source:HGNC Symbol;Acc:HGNC:7967] | 8481 | -0.192 | -0.2928 | No |
| 235 | PRDM5 | PR/SET domain 5 [Source:HGNC Symbol;Acc:HGNC:9349] | 8486 | -0.193 | -0.2917 | No |
| 236 | RNF6 | ring finger protein 6 [Source:HGNC Symbol;Acc:HGNC:10069] | 8493 | -0.193 | -0.2908 | No |
| 237 | LDB1 | LIM domain binding 1 [Source:HGNC Symbol;Acc:HGNC:6532] | 8522 | -0.195 | -0.2916 | No |
| 238 | NRIP1 | nuclear receptor interacting protein 1 [Source:HGNC Symbol;Acc:HGNC:8001] | 8524 | -0.195 | -0.2903 | No |
| 239 | CRY2 | cryptochrome circadian regulator 2 [Source:HGNC Symbol;Acc:HGNC:2385] | 8536 | -0.196 | -0.2898 | No |
| 240 | ATF2 | activating transcription factor 2 [Source:HGNC Symbol;Acc:HGNC:784] | 8592 | -0.201 | -0.2928 | No |
| 241 | KDM1A | lysine demethylase 1A [Source:HGNC Symbol;Acc:HGNC:29079] | 8608 | -0.202 | -0.2925 | No |
| 242 | ASXL1 | ASXL transcriptional regulator 1 [Source:HGNC Symbol;Acc:HGNC:18318] | 8610 | -0.202 | -0.2912 | No |
| 243 | CPNE1 | copine 1 [Source:HGNC Symbol;Acc:HGNC:2314] | 8642 | -0.204 | -0.2922 | No |
| 244 | AKAP8 | A-kinase anchoring protein 8 [Source:HGNC Symbol;Acc:HGNC:378] | 8659 | -0.205 | -0.2920 | No |
| 245 | RARG | retinoic acid receptor gamma [Source:HGNC Symbol;Acc:HGNC:9866] | 8700 | -0.209 | -0.2937 | No |
| 246 | DCAF1 | DDB1 and CUL4 associated factor 1 [Source:HGNC Symbol;Acc:HGNC:30911] | 8765 | -0.213 | -0.2973 | No |
| 247 | APEX1 | apurinic/apyrimidinic endodeoxyribonuclease 1 [Source:HGNC Symbol;Acc:HGNC:587] | 8767 | -0.213 | -0.2959 | No |
| 248 | RBPJ | recombination signal binding protein for immunoglobulin kappa J region [Source:HGNC Symbol;Acc:HGNC:5724] | 8800 | -0.217 | -0.2969 | No |
| 249 | TAF11 | TATA-box binding protein associated factor 11 [Source:HGNC Symbol;Acc:HGNC:11544] | 8860 | -0.222 | -0.3000 | No |
| 250 | TBX6 | T-box transcription factor 6 [Source:HGNC Symbol;Acc:HGNC:11605] | 8891 | -0.224 | -0.3008 | No |
| 251 | TLE1 | "TLE family member 1, transcriptional corepressor [Source:HGNC Symbol;Acc:HGNC:11837]" | 8895 | -0.224 | -0.2994 | No |
| 252 | CHD4 | chromodomain helicase DNA binding protein 4 [Source:HGNC Symbol;Acc:HGNC:1919] | 8917 | -0.226 | -0.2995 | No |
| 253 | TCF15 | transcription factor 15 [Source:HGNC Symbol;Acc:HGNC:11627] | 8925 | -0.226 | -0.2984 | No |
| 254 | DAXX | death domain associated protein [Source:HGNC Symbol;Acc:HGNC:2681] | 8926 | -0.226 | -0.2968 | No |
| 255 | SIRT1 | sirtuin 1 [Source:HGNC Symbol;Acc:HGNC:14929] | 8947 | -0.227 | -0.2968 | No |
| 256 | SPI1 | Spi-1 proto-oncogene [Source:HGNC Symbol;Acc:HGNC:11241] | 9012 | -0.232 | -0.3003 | No |
| 257 | SMAD4 | SMAD family member 4 [Source:HGNC Symbol;Acc:HGNC:6770] | 9035 | -0.235 | -0.3003 | No |
| 258 | HAND1 | heart and neural crest derivatives expressed 1 [Source:HGNC Symbol;Acc:HGNC:4807] | 9082 | -0.238 | -0.3023 | No |
| 259 | CIITA | class II major histocompatibility complex transactivator [Source:HGNC Symbol;Acc:HGNC:7067] | 9184 | -0.246 | -0.3086 | No |
| 260 | FKBP4 | FKBP prolyl isomerase 4 [Source:HGNC Symbol;Acc:HGNC:3720] | 9205 | -0.248 | -0.3085 | No |
| 261 | LCOR | ligand dependent nuclear receptor corepressor [Source:HGNC Symbol;Acc:HGNC:29503] | 9227 | -0.250 | -0.3084 | No |
| 262 | BCL10 | BCL10 immune signaling adaptor [Source:HGNC Symbol;Acc:HGNC:989] | 9248 | -0.252 | -0.3082 | No |
| 263 | AHR | aryl hydrocarbon receptor [Source:HGNC Symbol;Acc:HGNC:348] | 9263 | -0.253 | -0.3075 | No |
| 264 | NCOA1 | nuclear receptor coactivator 1 [Source:HGNC Symbol;Acc:HGNC:7668] | 9270 | -0.253 | -0.3061 | No |
| 265 | POU4F1 | POU class 4 homeobox 1 [Source:HGNC Symbol;Acc:HGNC:9218] | 9300 | -0.256 | -0.3066 | No |
| 266 | HDAC2 | histone deacetylase 2 [Source:HGNC Symbol;Acc:HGNC:4853] | 9359 | -0.260 | -0.3094 | No |
| 267 | CRX | cone-rod homeobox [Source:HGNC Symbol;Acc:HGNC:2383] | 9397 | -0.263 | -0.3105 | No |
| 268 | PAGR1 | PAXIP1 associated glutamate rich protein 1 [Source:HGNC Symbol;Acc:HGNC:28707] | 9401 | -0.264 | -0.3088 | No |
| 269 | ARNT | aryl hydrocarbon receptor nuclear translocator [Source:HGNC Symbol;Acc:HGNC:700] | 9427 | -0.267 | -0.3089 | No |
| 270 | GTF2H1 | general transcription factor IIH subunit 1 [Source:HGNC Symbol;Acc:HGNC:4655] | 9455 | -0.269 | -0.3092 | No |
| 271 | NKX2-1 | NK2 homeobox 1 [Source:HGNC Symbol;Acc:HGNC:11825] | 9480 | -0.272 | -0.3091 | No |
| 272 | PRDM13 | PR/SET domain 13 [Source:HGNC Symbol;Acc:HGNC:13998] | 9484 | -0.273 | -0.3074 | No |
| 273 | DDIT3 | DNA damage inducible transcript 3 [Source:HGNC Symbol;Acc:HGNC:2726] | 9551 | -0.278 | -0.3107 | No |
| 274 | TACC2 | transforming acidic coiled-coil containing protein 2 [Source:HGNC Symbol;Acc:HGNC:11523] | 9581 | -0.281 | -0.3110 | No |
| 275 | PRKDC | "protein kinase, DNA-activated, catalytic subunit [Source:HGNC Symbol;Acc:HGNC:9413]" | 9632 | -0.286 | -0.3130 | No |
| 276 | TACC1 | transforming acidic coiled-coil containing protein 1 [Source:HGNC Symbol;Acc:HGNC:11522] | 9690 | -0.290 | -0.3155 | No |
| 277 | EIF4E | eukaryotic translation initiation factor 4E [Source:HGNC Symbol;Acc:HGNC:3287] | 9709 | -0.293 | -0.3148 | No |
| 278 | CRTC1 | CREB regulated transcription coactivator 1 [Source:HGNC Symbol;Acc:HGNC:16062] | 9718 | -0.294 | -0.3134 | No |
| 279 | DDRGK1 | DDRGK domain containing 1 [Source:HGNC Symbol;Acc:HGNC:16110] | 9741 | -0.296 | -0.3130 | No |
| 280 | ETS1 | "ETS proto-oncogene 1, transcription factor [Source:HGNC Symbol;Acc:HGNC:3488]" | 9795 | -0.301 | -0.3151 | No |
| 281 | KAT2A | lysine acetyltransferase 2A [Source:HGNC Symbol;Acc:HGNC:4201] | 9799 | -0.301 | -0.3132 | No |
| 282 | PBX2 | PBX homeobox 2 [Source:HGNC Symbol;Acc:HGNC:8633] | 9804 | -0.302 | -0.3113 | No |
| 283 | NKX2-5 | NK2 homeobox 5 [Source:HGNC Symbol;Acc:HGNC:2488] | 9875 | -0.309 | -0.3147 | No |
| 284 | MLXIPL | MLX interacting protein like [Source:HGNC Symbol;Acc:HGNC:12744] | 9882 | -0.310 | -0.3130 | No |
| 285 | FOXH1 | forkhead box H1 [Source:HGNC Symbol;Acc:HGNC:3814] | 9884 | -0.310 | -0.3109 | No |
| 286 | TRIM24 | tripartite motif containing 24 [Source:HGNC Symbol;Acc:HGNC:11812] | 9927 | -0.314 | -0.3120 | No |
| 287 | HMGA1 | high mobility group AT-hook 1 [Source:HGNC Symbol;Acc:HGNC:5010] | 9966 | -0.318 | -0.3127 | No |
| 288 | FLNA | filamin A [Source:HGNC Symbol;Acc:HGNC:3754] | 9976 | -0.318 | -0.3112 | No |
| 289 | SMARCA4 | "SWI/SNF related, matrix associated, actin dependent regulator of chromatin, subfamily a, member 4 [Source:HGNC Symbol;Acc:HGNC:11100]" | 10009 | -0.322 | -0.3114 | No |
| 290 | TBXT | T-box transcription factor T [Source:HGNC Symbol;Acc:HGNC:11515] | 10034 | -0.325 | -0.3110 | No |
| 291 | KDM4C | lysine demethylase 4C [Source:HGNC Symbol;Acc:HGNC:17071] | 10040 | -0.325 | -0.3091 | No |
| 292 | DNAJA1 | DnaJ heat shock protein family (Hsp40) member A1 [Source:HGNC Symbol;Acc:HGNC:5229] | 10041 | -0.325 | -0.3068 | No |
| 293 | SMARCB1 | "SWI/SNF related, matrix associated, actin dependent regulator of chromatin, subfamily b, member 1 [Source:HGNC Symbol;Acc:HGNC:11103]" | 10117 | -0.333 | -0.3104 | No |
| 294 | CTBP1 | C-terminal binding protein 1 [Source:HGNC Symbol;Acc:HGNC:2494] | 10188 | -0.339 | -0.3136 | No |
| 295 | PRDM16 | PR/SET domain 16 [Source:HGNC Symbol;Acc:HGNC:14000] | 10211 | -0.342 | -0.3129 | No |
| 296 | ACTN4 | actinin alpha 4 [Source:HGNC Symbol;Acc:HGNC:166] | 10472 | -0.370 | -0.3311 | No |
| 297 | PPARGC1A | PPARG coactivator 1 alpha [Source:HGNC Symbol;Acc:HGNC:9237] | 10504 | -0.374 | -0.3309 | No |
| 298 | MTA2 | metastasis associated 1 family member 2 [Source:HGNC Symbol;Acc:HGNC:7411] | 10600 | -0.383 | -0.3357 | Yes |
| 299 | EXOSC9 | exosome component 9 [Source:HGNC Symbol;Acc:HGNC:9137] | 10606 | -0.384 | -0.3334 | Yes |
| 300 | DHX9 | DExH-box helicase 9 [Source:HGNC Symbol;Acc:HGNC:2750] | 10627 | -0.386 | -0.3322 | Yes |
| 301 | FOS | "Fos proto-oncogene, AP-1 transcription factor subunit [Source:HGNC Symbol;Acc:HGNC:3796]" | 10640 | -0.387 | -0.3304 | Yes |
| 302 | NOTCH2 | notch receptor 2 [Source:HGNC Symbol;Acc:HGNC:7882] | 10720 | -0.395 | -0.3339 | Yes |
| 303 | TDG | thymine DNA glycosylase [Source:HGNC Symbol;Acc:HGNC:11700] | 10728 | -0.396 | -0.3316 | Yes |
| 304 | BEX1 | brain expressed X-linked 1 [Source:HGNC Symbol;Acc:HGNC:1036] | 10772 | -0.402 | -0.3322 | Yes |
| 305 | IKZF4 | IKAROS family zinc finger 4 [Source:HGNC Symbol;Acc:HGNC:13179] | 10792 | -0.404 | -0.3308 | Yes |
| 306 | TXLNG | taxilin gamma [Source:HGNC Symbol;Acc:HGNC:18578] | 10828 | -0.408 | -0.3307 | Yes |
| 307 | SOX17 | SRY-box transcription factor 17 [Source:HGNC Symbol;Acc:HGNC:18122] | 10843 | -0.410 | -0.3289 | Yes |
| 308 | CREBBP | CREB binding protein [Source:HGNC Symbol;Acc:HGNC:2348] | 10881 | -0.414 | -0.3289 | Yes |
| 309 | GRIP1 | glutamate receptor interacting protein 1 [Source:HGNC Symbol;Acc:HGNC:18708] | 10949 | -0.424 | -0.3312 | Yes |
| 310 | GATA1 | GATA binding protein 1 [Source:HGNC Symbol;Acc:HGNC:4170] | 10958 | -0.425 | -0.3288 | Yes |
| 311 | ZBTB17 | zinc finger and BTB domain containing 17 [Source:HGNC Symbol;Acc:HGNC:12936] | 10996 | -0.429 | -0.3287 | Yes |
| 312 | RNF25 | ring finger protein 25 [Source:HGNC Symbol;Acc:HGNC:14662] | 11015 | -0.432 | -0.3271 | Yes |
| 313 | ETS2 | "ETS proto-oncogene 2, transcription factor [Source:HGNC Symbol;Acc:HGNC:3489]" | 11057 | -0.439 | -0.3272 | Yes |
| 314 | GATA2 | GATA binding protein 2 [Source:HGNC Symbol;Acc:HGNC:4171] | 11058 | -0.439 | -0.3241 | Yes |
| 315 | STK4 | serine/threonine kinase 4 [Source:HGNC Symbol;Acc:HGNC:11408] | 11074 | -0.441 | -0.3221 | Yes |
| 316 | PPARA | peroxisome proliferator activated receptor alpha [Source:HGNC Symbol;Acc:HGNC:9232] | 11110 | -0.445 | -0.3218 | Yes |
| 317 | TBP | TATA-box binding protein [Source:HGNC Symbol;Acc:HGNC:11588] | 11138 | -0.448 | -0.3207 | Yes |
| 318 | MED1 | mediator complex subunit 1 [Source:HGNC Symbol;Acc:HGNC:9234] | 11146 | -0.450 | -0.3181 | Yes |
| 319 | MEF2D | myocyte enhancer factor 2D [Source:HGNC Symbol;Acc:HGNC:6997] | 11149 | -0.451 | -0.3150 | Yes |
| 320 | GSK3B | glycogen synthase kinase 3 beta [Source:HGNC Symbol;Acc:HGNC:4617] | 11157 | -0.451 | -0.3123 | Yes |
| 321 | HSF1 | heat shock transcription factor 1 [Source:HGNC Symbol;Acc:HGNC:5224] | 11189 | -0.456 | -0.3116 | Yes |
| 322 | RPS3 | ribosomal protein S3 [Source:HGNC Symbol;Acc:HGNC:10420] | 11245 | -0.463 | -0.3126 | Yes |
| 323 | CRY1 | cryptochrome circadian regulator 1 [Source:HGNC Symbol;Acc:HGNC:2384] | 11247 | -0.463 | -0.3094 | Yes |
| 324 | TBL1X | transducin beta like 1 X-linked [Source:HGNC Symbol;Acc:HGNC:11585] | 11263 | -0.466 | -0.3073 | Yes |
| 325 | PTPN2 | protein tyrosine phosphatase non-receptor type 2 [Source:HGNC Symbol;Acc:HGNC:9650] | 11270 | -0.467 | -0.3044 | Yes |
| 326 | PIAS2 | protein inhibitor of activated STAT 2 [Source:HGNC Symbol;Acc:HGNC:17311] | 11288 | -0.470 | -0.3024 | Yes |
| 327 | PPARD | peroxisome proliferator activated receptor delta [Source:HGNC Symbol;Acc:HGNC:9235] | 11301 | -0.471 | -0.3000 | Yes |
| 328 | TMF1 | TATA element modulatory factor 1 [Source:HGNC Symbol;Acc:HGNC:11870] | 11328 | -0.475 | -0.2987 | Yes |
| 329 | NKX3-1 | NK3 homeobox 1 [Source:HGNC Symbol;Acc:HGNC:7838] | 11348 | -0.478 | -0.2968 | Yes |
| 330 | PIAS1 | protein inhibitor of activated STAT 1 [Source:HGNC Symbol;Acc:HGNC:2752] | 11422 | -0.490 | -0.2991 | Yes |
| 331 | BCL3 | BCL3 transcription coactivator [Source:HGNC Symbol;Acc:HGNC:998] | 11431 | -0.493 | -0.2963 | Yes |
| 332 | SETD6 | "SET domain containing 6, protein lysine methyltransferase [Source:HGNC Symbol;Acc:HGNC:26116]" | 11467 | -0.498 | -0.2955 | Yes |
| 333 | BRD8 | bromodomain containing 8 [Source:HGNC Symbol;Acc:HGNC:19874] | 11557 | -0.508 | -0.2990 | Yes |
| 334 | ZBTB7A | zinc finger and BTB domain containing 7A [Source:HGNC Symbol;Acc:HGNC:18078] | 11600 | -0.516 | -0.2987 | Yes |
| 335 | TWIST1 | twist family bHLH transcription factor 1 [Source:HGNC Symbol;Acc:HGNC:12428] | 11696 | -0.531 | -0.3025 | Yes |
| 336 | NR4A1 | nuclear receptor subfamily 4 group A member 1 [Source:HGNC Symbol;Acc:HGNC:7980] | 11709 | -0.535 | -0.2996 | Yes |
| 337 | TBX2 | T-box transcription factor 2 [Source:HGNC Symbol;Acc:HGNC:11597] | 11728 | -0.538 | -0.2972 | Yes |
| 338 | TCP10L | t-complex 10 like [Source:HGNC Symbol;Acc:HGNC:11657] | 11731 | -0.538 | -0.2935 | Yes |
| 339 | EP300 | E1A binding protein p300 [Source:HGNC Symbol;Acc:HGNC:3373] | 11741 | -0.540 | -0.2904 | Yes |
| 340 | MAP3K10 | mitogen-activated protein kinase kinase kinase 10 [Source:HGNC Symbol;Acc:HGNC:6849] | 11753 | -0.541 | -0.2874 | Yes |
| 341 | EPAS1 | endothelial PAS domain protein 1 [Source:HGNC Symbol;Acc:HGNC:3374] | 11758 | -0.542 | -0.2838 | Yes |
| 342 | LATS1 | large tumor suppressor kinase 1 [Source:HGNC Symbol;Acc:HGNC:6514] | 11760 | -0.542 | -0.2800 | Yes |
| 343 | CTDP1 | CTD phosphatase subunit 1 [Source:HGNC Symbol;Acc:HGNC:2498] | 11805 | -0.550 | -0.2796 | Yes |
| 344 | HIF1A | hypoxia inducible factor 1 subunit alpha [Source:HGNC Symbol;Acc:HGNC:4910] | 11833 | -0.556 | -0.2778 | Yes |
| 345 | PDCD11 | programmed cell death 11 [Source:HGNC Symbol;Acc:HGNC:13408] | 11847 | -0.559 | -0.2748 | Yes |
| 346 | ID2 | inhibitor of DNA binding 2 [Source:HGNC Symbol;Acc:HGNC:5361] | 11852 | -0.560 | -0.2711 | Yes |
| 347 | TRIB1 | tribbles pseudokinase 1 [Source:HGNC Symbol;Acc:HGNC:16891] | 11924 | -0.577 | -0.2727 | Yes |
| 348 | NUCKS1 | nuclear casein kinase and cyclin dependent kinase substrate 1 [Source:HGNC Symbol;Acc:HGNC:29923] | 11945 | -0.581 | -0.2701 | Yes |
| 349 | TCERG1 | transcription elongation regulator 1 [Source:HGNC Symbol;Acc:HGNC:15630] | 11950 | -0.583 | -0.2663 | Yes |
| 350 | DDX54 | DEAD-box helicase 54 [Source:HGNC Symbol;Acc:HGNC:20084] | 11969 | -0.587 | -0.2635 | Yes |
| 351 | ATF4 | activating transcription factor 4 [Source:HGNC Symbol;Acc:HGNC:786] | 11999 | -0.593 | -0.2616 | Yes |
| 352 | SPEN | spen family transcriptional repressor [Source:HGNC Symbol;Acc:HGNC:17575] | 12108 | -0.622 | -0.2658 | Yes |
| 353 | ARNTL | aryl hydrocarbon receptor nuclear translocator like [Source:HGNC Symbol;Acc:HGNC:701] | 12120 | -0.625 | -0.2622 | Yes |
| 354 | HIRA | histone cell cycle regulator [Source:HGNC Symbol;Acc:HGNC:4916] | 12143 | -0.630 | -0.2595 | Yes |
| 355 | HMGB1 | high mobility group box 1 [Source:HGNC Symbol;Acc:HGNC:4983] | 12170 | -0.636 | -0.2570 | Yes |
| 356 | TEAD3 | TEA domain transcription factor 3 [Source:HGNC Symbol;Acc:HGNC:11716] | 12178 | -0.637 | -0.2530 | Yes |
| 357 | DAPK3 | death associated protein kinase 3 [Source:HGNC Symbol;Acc:HGNC:2676] | 12180 | -0.639 | -0.2485 | Yes |
| 358 | ZMYND8 | zinc finger MYND-type containing 8 [Source:HGNC Symbol;Acc:HGNC:9397] | 12214 | -0.649 | -0.2465 | Yes |
| 359 | YY1 | YY1 transcription factor [Source:HGNC Symbol;Acc:HGNC:12856] | 12229 | -0.653 | -0.2430 | Yes |
| 360 | FOXO4 | forkhead box O4 [Source:HGNC Symbol;Acc:HGNC:7139] | 12240 | -0.656 | -0.2391 | Yes |
| 361 | NCL | nucleolin [Source:HGNC Symbol;Acc:HGNC:7667] | 12255 | -0.662 | -0.2355 | Yes |
| 362 | NOC2L | NOC2 like nucleolar associated transcriptional repressor [Source:HGNC Symbol;Acc:HGNC:24517] | 12258 | -0.663 | -0.2309 | Yes |
| 363 | GATA3 | GATA binding protein 3 [Source:HGNC Symbol;Acc:HGNC:4172] | 12276 | -0.670 | -0.2275 | Yes |
| 364 | NFIA | nuclear factor I A [Source:HGNC Symbol;Acc:HGNC:7784] | 12294 | -0.675 | -0.2240 | Yes |
| 365 | NR4A2 | nuclear receptor subfamily 4 group A member 2 [Source:HGNC Symbol;Acc:HGNC:7981] | 12368 | -0.698 | -0.2248 | Yes |
| 366 | REST | RE1 silencing transcription factor [Source:HGNC Symbol;Acc:HGNC:9966] | 12382 | -0.703 | -0.2209 | Yes |
| 367 | THRAP3 | thyroid hormone receptor associated protein 3 [Source:HGNC Symbol;Acc:HGNC:22964] | 12426 | -0.717 | -0.2192 | Yes |
| 368 | STRN | striatin [Source:HGNC Symbol;Acc:HGNC:11424] | 12429 | -0.717 | -0.2142 | Yes |
| 369 | NCOA3 | nuclear receptor coactivator 3 [Source:HGNC Symbol;Acc:HGNC:7670] | 12455 | -0.727 | -0.2110 | Yes |
| 370 | MED25 | mediator complex subunit 25 [Source:HGNC Symbol;Acc:HGNC:28845] | 12480 | -0.741 | -0.2076 | Yes |
| 371 | PER1 | period circadian regulator 1 [Source:HGNC Symbol;Acc:HGNC:8845] | 12496 | -0.746 | -0.2035 | Yes |
| 372 | MED13 | mediator complex subunit 13 [Source:HGNC Symbol;Acc:HGNC:22474] | 12504 | -0.749 | -0.1987 | Yes |
| 373 | CCNT1 | cyclin T1 [Source:HGNC Symbol;Acc:HGNC:1599] | 12517 | -0.757 | -0.1942 | Yes |
| 374 | NPM1 | nucleophosmin 1 [Source:HGNC Symbol;Acc:HGNC:7910] | 12530 | -0.762 | -0.1898 | Yes |
| 375 | NR4A3 | nuclear receptor subfamily 4 group A member 3 [Source:HGNC Symbol;Acc:HGNC:7982] | 12543 | -0.769 | -0.1852 | Yes |
| 376 | SRF | serum response factor [Source:HGNC Symbol;Acc:HGNC:11291] | 12549 | -0.773 | -0.1801 | Yes |
| 377 | SP1 | Sp1 transcription factor [Source:HGNC Symbol;Acc:HGNC:11205] | 12557 | -0.777 | -0.1751 | Yes |
| 378 | BHLHE40 | basic helix-loop-helix family member e40 [Source:HGNC Symbol;Acc:HGNC:1046] | 12573 | -0.782 | -0.1707 | Yes |
| 379 | KDM5D | lysine demethylase 5D [Source:HGNC Symbol;Acc:HGNC:11115] | 12595 | -0.793 | -0.1667 | Yes |
| 380 | HDAC11 | histone deacetylase 11 [Source:HGNC Symbol;Acc:HGNC:19086] | 12601 | -0.799 | -0.1614 | Yes |
| 381 | GABARAPL1 | GABA type A receptor associated protein like 1 [Source:HGNC Symbol;Acc:HGNC:4068] | 12616 | -0.808 | -0.1567 | Yes |
| 382 | MTDH | metadherin [Source:HGNC Symbol;Acc:HGNC:29608] | 12621 | -0.809 | -0.1513 | Yes |
| 383 | BCL6 | BCL6 transcription repressor [Source:HGNC Symbol;Acc:HGNC:1001] | 12667 | -0.838 | -0.1489 | Yes |
| 384 | KLF5 | Kruppel like factor 5 [Source:HGNC Symbol;Acc:HGNC:6349] | 12672 | -0.845 | -0.1431 | Yes |
| 385 | HES1 | hes family bHLH transcription factor 1 [Source:HGNC Symbol;Acc:HGNC:5192] | 12676 | -0.849 | -0.1373 | Yes |
| 386 | ARID5A | AT-rich interaction domain 5A [Source:HGNC Symbol;Acc:HGNC:17361] | 12705 | -0.867 | -0.1334 | Yes |
| 387 | CGGBP1 | CGG triplet repeat binding protein 1 [Source:HGNC Symbol;Acc:HGNC:1888] | 12722 | -0.883 | -0.1283 | Yes |
| 388 | PTMA | prothymosin alpha [Source:HGNC Symbol;Acc:HGNC:9623] | 12742 | -0.906 | -0.1234 | Yes |
| 389 | ACTB | actin beta [Source:HGNC Symbol;Acc:HGNC:132] | 12757 | -0.920 | -0.1179 | Yes |
| 390 | MTA1 | metastasis associated 1 [Source:HGNC Symbol;Acc:HGNC:7410] | 12792 | -0.952 | -0.1138 | Yes |
| 391 | SETD1A | "SET domain containing 1A, histone lysine methyltransferase [Source:HGNC Symbol;Acc:HGNC:29010]" | 12793 | -0.954 | -0.1070 | Yes |
| 392 | MYC | "MYC proto-oncogene, bHLH transcription factor [Source:HGNC Symbol;Acc:HGNC:7553]" | 12801 | -0.970 | -0.1006 | Yes |
| 393 | CEBPB | CCAAT enhancer binding protein beta [Source:HGNC Symbol;Acc:HGNC:1834] | 12804 | -0.973 | -0.0938 | Yes |
| 394 | JUN | "Jun proto-oncogene, AP-1 transcription factor subunit [Source:HGNC Symbol;Acc:HGNC:6204]" | 12813 | -0.994 | -0.0873 | Yes |
| 395 | NFKBIA | NFKB inhibitor alpha [Source:HGNC Symbol;Acc:HGNC:7797] | 12817 | -1.001 | -0.0804 | Yes |
| 396 | DDX5 | DEAD-box helicase 5 [Source:HGNC Symbol;Acc:HGNC:2746] | 12834 | -1.038 | -0.0743 | Yes |
| 397 | TOB2 | "transducer of ERBB2, 2 [Source:HGNC Symbol;Acc:HGNC:11980]" | 12838 | -1.041 | -0.0671 | Yes |
| 398 | TPT1 | "tumor protein, translationally-controlled 1 [Source:HGNC Symbol;Acc:HGNC:12022]" | 12839 | -1.041 | -0.0596 | Yes |
| 399 | HIPK2 | homeodomain interacting protein kinase 2 [Source:HGNC Symbol;Acc:HGNC:14402] | 12842 | -1.045 | -0.0523 | Yes |
| 400 | KLF4 | Kruppel like factor 4 [Source:HGNC Symbol;Acc:HGNC:6348] | 12848 | -1.058 | -0.0452 | Yes |
| 401 | SIK1 | salt inducible kinase 1 [Source:HGNC Symbol;Acc:HGNC:11142] | 12863 | -1.104 | -0.0384 | Yes |
| 402 | JUND | "JunD proto-oncogene, AP-1 transcription factor subunit [Source:HGNC Symbol;Acc:HGNC:6206]" | 12865 | -1.116 | -0.0305 | Yes |
| 403 | CTNNB1 | catenin beta 1 [Source:HGNC Symbol;Acc:HGNC:2514] | 12866 | -1.119 | -0.0225 | Yes |
| 404 | HDAC7 | histone deacetylase 7 [Source:HGNC Symbol;Acc:HGNC:14067] | 12870 | -1.131 | -0.0146 | Yes |
| 405 | BAZ2A | bromodomain adjacent to zinc finger domain 2A [Source:HGNC Symbol;Acc:HGNC:962] | 12879 | -1.166 | -0.0069 | Yes |
| 406 | TAF1 | TATA-box binding protein associated factor 1 [Source:HGNC Symbol;Acc:HGNC:11535] | 12881 | -1.192 | 0.0015 | Yes |
Table: GSEA details [plain text format]

  

Fig 2: GOMF\_DNA\_BINDING\_TRANSCRIPTION\_FACTOR\_BINDING      
 Blue-Pink O' Gram in the Space of the Analyzed GeneSet

  

Fig 3: GOMF\_DNA\_BINDING\_TRANSCRIPTION\_FACTOR\_BINDING: Random ES distribution      
 Gene set null distribution of ES for **GOMF\_DNA\_BINDING\_TRANSCRIPTION\_FACTOR\_BINDING**
